# Patient-Reported Pain and Satisfaction During Flexible Cystoscopy in Supine Versus Lithotomy Positions in Adult Men: A Retrospective Observational Study

**DOI:** 10.3390/healthcare14162652

**Published:** 2026-08-21

**Authors:** Erbay Tumer, Eyup Kaplan

**Affiliations:** 1 Department of Urology, NCR International Hospital, 27060 Gaziantep, Turkey; 2 Department of Urology, Gaziantep Islam Science and Technology University, 27010 Gaziantep, Turkey; dreyup001@hotmail.com

**Keywords:** flexible cystoscopy, supine position, lithotomy position, pain, patient satisfaction, VAS, urology, endoscopy

## Abstract

Background: Supine flexible cystoscopy is already established in routine urological practice. However, real-world patient-reported pain across specific procedural stages and other patient-centered outcomes according to clinician-selected procedural position remain incompletely characterized. This study evaluated the associations of supine and lithotomy positioning with patient-reported and procedural outcomes in adult men undergoing flexible cystoscopy. Materials and Methods: This multicenter retrospective observational study included 80 adult male patients who underwent flexible cystoscopy under local anesthesia. Procedural position was selected by the treating urologist according to clinical feasibility and routine clinical judgment, including the patient’s ability to assume the lithotomy position. Patients were classified according to the position used during the procedure as supine (*n* = 40) or lithotomy (*n* = 40). Pain was assessed using a 0–10 Visual Analog Scale (VAS), with higher scores indicating greater pain. Patient satisfaction was assessed using a separate 0–10 scale. Secondary outcomes included procedure duration, procedural success, complication rates, and patient tolerance. Procedural tolerance and overall patient satisfaction were assessed immediately after cystoscopy using separate single-item 0–10 rating scales. For the satisfaction scale, 0 indicated “completely dissatisfied” and 10 indicated “completely satisfied”. Group comparisons and an exploratory multivariable linear regression analysis of the primary pain outcome were performed. Results: A total of 80 patients were included (40 supine and 40 lithotomy). Mean VAS scores were lower in the supine group during cystoscope insertion (2.1 ± 1.0 vs. 3.4 ± 1.2; mean difference [MD]: −1.30, 95% CI: −1.79 to −0.81; *p* < 0.001), bladder evaluation (1.8 ± 0.9 vs. 2.6 ± 1.1; MD: −0.80, 95% CI: −1.25 to −0.35; *p* = 0.001), and immediately after the procedure (1.2 ± 0.7 vs. 2.0 ± 0.9; MD: −0.80, 95% CI: −1.16 to −0.44; *p* < 0.001). Procedure duration was 6.8 ± 2.1 versus 7.4 ± 2.3 min (MD: −0.60 min, 95% CI: −1.58 to 0.38; *p* = 0.186). Procedural success occurred in 97.5% versus 95.0% of patients (risk difference [RD]: 2.5 percentage points, 95% CI: −8.5 to 14.2; *p* = 1.000). In an exploratory post hoc subgroup of patients with documented difficult catheterization (*n* = 40; 20 patients per group), cystoscopy-guided catheterization was successful in 19/20 patients (95.0%) in the supine group and 13/20 patients (65.0%) in the lithotomy group (RD: 30.0 percentage points, 95% CI: 4.9 to 52.1; *p* = 0.044). This small, non-prespecified subgroup analysis was considered hypothesis-generating. Complications occurred in 4/40 patients (10.0%) in the supine group and 7/40 patients (17.5%) in the lithotomy group (RD: −7.5 percentage points, 95% CI: −23.2 to 8.2; two-sided Fisher’s exact *p* = 0.518). Procedural tolerance (8.6 ± 1.1 vs. 7.2 ± 1.4; MD: 1.40, 95% CI: 0.84 to 1.96; *p* < 0.001) and overall satisfaction scores (8.8 ± 1.0 vs. 7.4 ± 1.3; MD: 1.40, 95% CI: 0.88 to 1.92; *p* < 0.001) were higher in the supine group. After adjustment for the available covariates, supine positioning remained associated with a lower insertion-related VAS score (β = −1.21, 95% CI: −1.75 to −0.67; *p* < 0.001). Conclusions: In this retrospective, non-randomized cohort, clinician-selected supine positioning was associated with lower reported pain and higher procedural tolerance and satisfaction compared with lithotomy positioning. No statistically significant between-group differences were observed in overall procedural success or complication rates. Because position selection was clinician-directed and potentially influenced by patient mobility and other unmeasured factors, these findings do not demonstrate a causal benefit of supine positioning. They should be considered hypothesis-generating and require confirmation in adequately powered prospective randomized studies before informing changes in routine clinical practice.

## 1. Introduction

Cystoscopy is one of the fundamental diagnostic and therapeutic procedures in urological practice, allowing direct endoscopic visualization of the urethra, prostate, bladder neck, and urinary bladder. It is widely used in the evaluation of hematuria, lower urinary tract symptoms, urethral strictures, bladder outlet obstruction, foreign bodies, and the diagnosis and surveillance of bladder tumors [1,2,3]. In contemporary urology, flexible cystoscopy has increasingly replaced rigid cystoscopy in many outpatient and bedside settings because it is less invasive, can be performed under local anesthesia, and is generally associated with better patient tolerance [4].

Both supine and lithotomy positioning may be used during flexible cystoscopy. Although lithotomy positioning provides familiar procedural access, it requires hip and knee flexion with leg elevation and abduction, which may be uncomfortable or impractical in patients with restricted mobility, musculoskeletal disease, contractures, obesity, neurological impairment, or critical illness [5,6,7,8,9,10]. Supine positioning avoids leg elevation and may therefore offer a practical option when assuming or maintaining lithotomy is difficult.

Flexible cystoscopy permits urethral and bladder evaluation with minimal patient repositioning and can be performed in outpatient or bedside settings. When combined with intraurethral local anesthetic gel, it generally does not require general or spinal anesthesia [11], making supine positioning a practical option when lithotomy positioning or operating room transfer is difficult.

Pain and comfort are important determinants of cystoscopy acceptability, and flexible cystoscopy is generally associated with less discomfort than rigid cystoscopy [12,13,14]. Pain perception is nevertheless multifactorial and may be influenced by urethral anatomy, anxiety, previous procedural experience, instrument and anesthetic characteristics, procedure duration, and patient position. In patients with restricted mobility, maintaining lithotomy and leg elevation may add discomfort, whereas supine positioning avoids these requirements.

Although a recent randomized controlled trial reported lower pain and shorter procedure duration with supine positioning [15], complementary observational data can characterize clinician-directed position selection and its associations with stage-specific pain and broader patient-centered and procedural outcomes in a consecutive real-world cohort. Flexible cystoscopy can facilitate guidewire-assisted catheter placement under direct visualization after failed conventional catheterization and may be particularly practical in the supine position for patients unable to assume lithotomy. However, direct comparative evidence regarding pain, procedural outcomes, tolerance, and satisfaction between supine and lithotomy flexible cystoscopy remains limited. Evaluating these outcomes may provide preliminary evidence regarding the feasibility of supine cystoscopy, although findings from non-randomized comparisons require cautious interpretation.

This retrospective observational study aimed to compare patient-reported pain during flexible cystoscopy performed under local anesthesia in the supine and lithotomy positions in adult male patients. The insertion-related Visual Analog Scale score was the primary outcome, while secondary outcomes included procedure duration, procedural success, complications, patient-reported procedural tolerance, and overall satisfaction. Given the non-randomized design, the study evaluated associations between procedural position and these outcomes rather than seeking to establish the causal superiority of either position.

## 2. Materials and Methods

### 2.1. Study Design

The study protocol was reviewed and approved by the Gaziantep City Hospital Non-Interventional Clinical Research Ethics Committee (Decision No: 438/2026; Date: 18 February 2026). The study was conducted in accordance with the principles of the Declaration of Helsinki. Although the study was retrospective, written informed consent for participation and the use of clinical data for research purposes was obtained from all included patients.

This study was designed as a multicenter, retrospective, observational, comparative study. The study was conducted at the Urology Outpatient Clinics of Gaziantep NCR International Hospital and Gaziantep Abdulkadir Yuksel Hospital between January 2024 and December 2025. The period from January 2024 to December 2025 represents the clinical procedure and routine documentation period. Retrospective screening of the medical records, research-specific data extraction, anonymization, and statistical analysis commenced only after ethics approval was obtained on 18 February 2026. The primary aim was to evaluate the association between procedural position (supine vs. lithotomy) and patient-reported and procedural outcomes during flexible cystoscopy performed under local anesthesia.

### 2.2. Study Population

Adult male patients (≥18 years) who underwent flexible cystoscopy under local anesthesia during the study period were retrospectively evaluated for eligibility. Consecutive eligible procedures with complete clinical and outcome data were included. Inclusion criteria comprised an indication for diagnostic cystoscopy, such as hematuria, lower urinary tract symptoms, or suspected urethral pathology; performance of flexible cystoscopy under local anesthesia; and performance of the procedure in either the supine or lithotomy position. Exclusion criteria included procedures performed under general or spinal anesthesia, use of rigid cystoscopy, active urinary tract infection at the time of the procedure, major intraprocedural complications necessitating termination of the procedure, and incomplete clinical or outcome data.

Procedural position was not randomly assigned and was selected by the treating urologist before the procedure according to clinical feasibility and routine clinical judgment. Factors considered included the patient’s mobility, ability to tolerate hip and knee flexion and leg elevation, musculoskeletal limitations, and the feasibility of assuming the conventional lithotomy position. Consequently, patients with limited mobility or difficulty assuming the lithotomy position were more likely to undergo cystoscopy in the supine position. The groups therefore reflect clinician-directed positioning in routine practice and are subject to potential selection bias and confounding by indication. Patients were classified according to the position actually used during the procedure:

- **Group 1 (Supine Group):** Patients who underwent flexible cystoscopy in the standard supine position.
- **Group 2 (Lithotomy Group):** Patients who underwent flexible cystoscopy in the conventional lithotomy position.

Position selection was made before the procedure and was not based on a formal institutional allocation protocol or standardized scoring system. In routine practice, the treating urologist considered whether the patient could safely and comfortably assume and maintain hip and knee flexion, leg abduction, and leg elevation in the lithotomy position. Following a secondary review of the source records, we additionally abstracted any explicit documentation of mobility or musculoskeletal limitations, difficulty assuming or maintaining lithotomy, preprocedural anxiety, previous cystoscopy experience, and the stated reason for position selection. Each factor was classified as present or absent only when the available documentation permitted such classification; otherwise, it was recorded as missing. Lack of documentation was not interpreted as absence of the characteristic.

Demographic characteristics, cystoscopy indications, recorded comorbidities, anticoagulant use, previous urological interventions, study center, anonymized operator identity, procedural characteristics, patient-reported outcomes, and the available position-selection variables were extracted from the medical records. Mobility limitation, preprocedural anxiety, baseline position-related discomfort, and previous cystoscopy experience had not been collected prospectively or assessed using standardized instruments. Accordingly, the available information on these factors was reported descriptively with its corresponding availability and was not incorporated into the primary adjusted model.

### 2.3. Procedure Technique

All procedures were performed using a Redpine single-use video flexible cystoscope system (RP-U-C01F, Guangzhou Red Pine Medical Instrument Co., Ltd., Guangzhou, China). Before cystoscope insertion, 10–15 mL of 2% intraurethral lidocaine gel was administered and retained for approximately 5–10 min. For routine diagnostic cystoscopy, the flexible cystoscope was advanced directly through the urethra without guidewire assistance. In patients with documented failure of conventional blind catheterization, a hydrophilic guidewire was advanced into the bladder through the working channel of the flexible cystoscope under direct visualization. After withdrawal of the cystoscope while maintaining guidewire access, a urethral catheter was advanced over the guidewire into the bladder. Successful placement was confirmed by urinary drainage. The cystoscope was used as part of routine institutional clinical practice. Neither the manufacturer nor its distributor participated in the study design, data collection, analysis, interpretation, or manuscript preparation.

Procedures were performed by experienced urologists as part of routine clinical practice; however, the operator distribution was not identical between the positional groups. Although the cystoscope system, local anesthetic protocol, and general procedural technique were standardized, residual confounding related to operator experience and technique cannot be excluded.

In the supine group, patients remained in the standard supine position on the examination table or hospital bed, without the need for repositioning or leg elevation. In the lithotomy group, patients were positioned in the standard lithotomy position with appropriate leg support.

All procedures were performed by experienced urologists using a standardized technique. Sterile conditions were maintained in all cases. The cystoscope was gently advanced under direct visualization through the urethra into the bladder. Diagnostic evaluation included inspection of the urethra, prostate, bladder neck, trigone, and bladder mucosa. In selected cases, minor therapeutic interventions such as guidewire-assisted urethral catheterization were performed under direct visualization.

Because this was a retrospective study, difficult catheterization was identified from the medical records rather than through prospective classification. For the present analysis, difficult catheterization was operationally defined as a documented failure of conventional blind transurethral catheterization followed by the need for flexible cystoscopy-guided catheter placement. Patients without explicit documentation of a failed conventional catheterization attempt were not classified as having difficult catheterization. Successful cystoscopy-guided catheterization was defined as successful advancement and placement of the urethral catheter into the bladder under direct cystoscopic guidance without requiring an alternative urinary drainage procedure. The difficult catheterization subgroup analysis was exploratory and post hoc. It was not prespecified in the study protocol or included in the original sample-size calculation, and no separate power calculation was performed for this subgroup.

### 2.4. Outcome Measures

The primary outcome was patient-reported pain during flexible cystoscopy, assessed using a 0–10 Visual Analog Scale (VAS), where 0 indicated no pain and 10 indicated the worst imaginable pain. Pain scores were obtained at three predefined time points: immediately after cystoscope insertion into the bladder, immediately after completion of the systematic bladder evaluation but before cystoscope withdrawal, and immediately after complete withdrawal of the cystoscope. At the first two time points, patients were instructed to rate the pain experienced during the corresponding procedural stage. The VAS score recorded during cystoscope insertion was used as the principal pain outcome in the adjusted analysis and was analyzed as a continuous variable. No categorical threshold was used to define high procedural pain.

Secondary outcomes included procedure duration, procedural success, complication rates, patient-reported procedural tolerance, and overall patient satisfaction. Immediately after the procedure, patients were asked two separate standardized questions: “How well were you able to physically tolerate the procedure?” and “Overall, how satisfied were you with your procedural experience?” Responses were recorded using separate single-item 0–10 rating scales. For procedural tolerance, 0 indicated “completely intolerable” and 10 indicated “completely tolerable.” For overall satisfaction, 0 indicated “completely dissatisfied” and 10 indicated “completely satisfied.” Both outcomes were analyzed as continuous variables, and no categorical threshold was used to define high satisfaction.

### 2.5. Pain Assessment, Patient-Reported Procedural Tolerance and Satisfaction

All patient-reported outcomes were obtained contemporaneously as part of routine institutional clinical practice and documented on the cystoscopy procedure record; they were not collected retrospectively through patient recall or specifically for the present study. A member of the routine urology clinical staff, typically the assisting nurse or clinical assistant present during the procedure, administered the rating instruments and recorded the responses. The operating urologist did not retrospectively assign or modify the patient-reported scores. Because routine staffing varied, the same staff member did not necessarily record the outcomes for all patients.

Pain was assessed using a 10 cm horizontal Visual Analog Scale (VAS) anchored by “no pain” at 0 and “the worst imaginable pain” at 10. Patients were asked to indicate their pain intensity on the scale at three predefined time points: immediately after passage of the cystoscope into the bladder, immediately after completion of the systematic bladder evaluation but before cystoscope withdrawal, and immediately after complete withdrawal of the cystoscope. The distance from the 0 anchor was recorded as a continuous score ranging from 0 to 10. The first and second assessments represented the pain experienced during cystoscope insertion and bladder evaluation, respectively. The insertion-related VAS score was designated as the primary pain outcome. The VAS is a widely used generic measure of pain intensity; however, it was not separately validated for flexible cystoscopy in the present cohort.

Procedural tolerance and overall satisfaction were assessed separately immediately after complete cystoscope withdrawal and before the patient left the procedure area. Patients were asked, “How well were you able to physically tolerate the procedure?” and “Overall, how satisfied were you with your procedural experience?” Responses were recorded using separate single-item 0–10 rating scales. For procedural tolerance, 0 indicated “completely intolerable” and 10 indicated “completely tolerable.” For overall satisfaction, 0 indicated “completely dissatisfied” and 10 indicated “completely satisfied.” These scales were used as part of institutional clinical practice and had not been formally validated as flexible-cystoscopy-specific instruments.

Procedural position was directly observable; therefore, patients, operating urologists, and clinical personnel administering and recording the outcome measures were not blinded to position. Data extraction from the medical records was also performed with knowledge of the documented procedural position. Consequently, response and observer-related measurement bias cannot be excluded.

### 2.6. Procedure Time, Definition of Procedural Success, and Complication Assessment

Procedure time was defined as the duration from insertion of the cystoscope into the urethral meatus to complete withdrawal of the instrument. Procedural success was defined as the completion of an adequate diagnostic cystoscopic evaluation and/or successful urethral catheterization in cases of difficult catheterization.

Complications were identified from the procedural records and documented clinical assessments during the early follow-up period of 24–48 h. Complications were categorized as urethral trauma, hematuria requiring additional clinical intervention, urinary tract infection, and acute urinary retention. “Any complication” was defined as the occurrence of at least one of these events and was analyzed as a patient-level composite outcome. Individual complication categories were not mutually exclusive; a patient with more than one complication was counted once in the composite outcome and once in each relevant complication category.

### 2.7. Sample Size Calculation

Sample size calculation was performed based on the primary endpoint, defined as the difference in Visual Analog Scale (VAS) pain scores between the two groups. According to previous studies, mean VAS scores during flexible cystoscopy range between 2.5 and 4.0, and patient positioning may significantly influence pain perception [15,16,17]. A clinically meaningful difference of 1.0 point in VAS score between groups was assumed, with a standard deviation (SD) of 1.5 for both groups. The sample size was calculated using G*Power software (version 3.1, Heinrich Heine University, Düsseldorf, Germany) based on a two-tailed independent samples t-test with a significance level (α) of 0.05 and a statistical power of 80% (1 − β = 0.80). The minimum required sample size was 36 patients per group (total *n* = 72). To account for potential exclusions and incomplete records, this target was increased by approximately 10% to 40 patients per group (total *n* = 80). All eligible records available during the predefined study period were screened, resulting in a final cohort of 80 patients. This calculation was intended to support the primary between-group comparison of VAS scores and was not designed to establish the stability of multivariable or predictive models.

### 2.8. Statistical Analysis

All statistical analyses were performed using IBM SPSS Statistics version 27.0 (IBM Corp., Armonk, NY, USA). Continuous variables were summarized as mean ± standard deviation or median with interquartile range, according to their distribution, and categorical variables were presented as frequencies and percentages. Distributional assumptions were evaluated using histograms, Q–Q plots, and the Kolmogorov–Smirnov test. Between-group comparisons were performed using the independent-samples t-test for normally distributed continuous variables and the Mann–Whitney U test for non-normally distributed variables. Categorical variables were compared using the chi-square test or two-sided Fisher’s exact test, as appropriate. Between-group effects for continuous outcomes were reported as mean differences with 95% confidence intervals. Categorical outcomes were summarized using absolute risk differences with 95% confidence intervals, calculated using the Newcombe method. Cohen’s d values were also reported with their corresponding 95% confidence intervals. Study-center and operator distributions were compared between the positional groups using Pearson’s chi-square test or Fisher’s exact test, as appropriate. Baseline characteristics were evaluated descriptively in addition to formal significance testing because the relatively small group sizes could limit the ability of *p*-values to identify clinically relevant imbalances. Two exploratory multivariable linear regression models were fitted. In the primary model, the dependent variable was the VAS score during cystoscope insertion, and the independent variables were procedural position (supine vs. lithotomy), age, BMI, anticoagulant use, and previous urological intervention. These covariates were selected on the basis of clinical relevance and their availability in the retrospective dataset and were entered simultaneously into the model without automated or stepwise variable selection. In the secondary model, overall patient satisfaction score was the dependent variable, and procedural position, VAS score during cystoscope insertion, and procedure duration were entered simultaneously as independent variables. Regression results were reported using unstandardized coefficients (B), standard errors (SEs), standardized coefficients (β), 95% confidence intervals (CIs), and *p*-values. Model fit was summarized using the sample size included in the model, R^2^, adjusted R^2^, and the overall F-test. The adjusted estimates represent conditional associations and should not be interpreted as causal effects. Covariate completeness was assessed before model fitting. Multicollinearity was assessed using tolerance and variance inflation factor (VIF) values before finalizing each regression model. A tolerance value <0.20 or a VIF > 5 was considered indicative of potentially important multicollinearity. Procedural position, age, BMI, anticoagulant use, previous urological intervention, insertion-related VAS score, procedure duration, and overall satisfaction score were available for all 80 included patients. Therefore, both regression models included the complete study cohort (*n* = 80), and no missing-data imputation was performed. The position-selection variables obtained during the secondary record review, including mobility limitation, preprocedural anxiety, and previous cystoscopy experience, were incompletely and non-standardly documented and were therefore reported descriptively but not included in the multivariable models. Because of the small sample size and low cell counts, cystoscopy-guided catheterization success in the exploratory post hoc difficult catheterization subgroup was compared using a two-sided Fisher’s exact test. No multivariable adjustment was performed for this subgroup. The analysis was not separately powered, and its results were interpreted as descriptive and hypothesis-generating. No prediction modeling, ROC analysis, calibration assessment, or decision curve analysis was performed. Secondary-outcome analyses were considered exploratory, and no adjustment for multiple comparisons was applied. A two-tailed *p*-value of <0.05 was considered statistically significant.

## 3. Results

**Figure 1:**
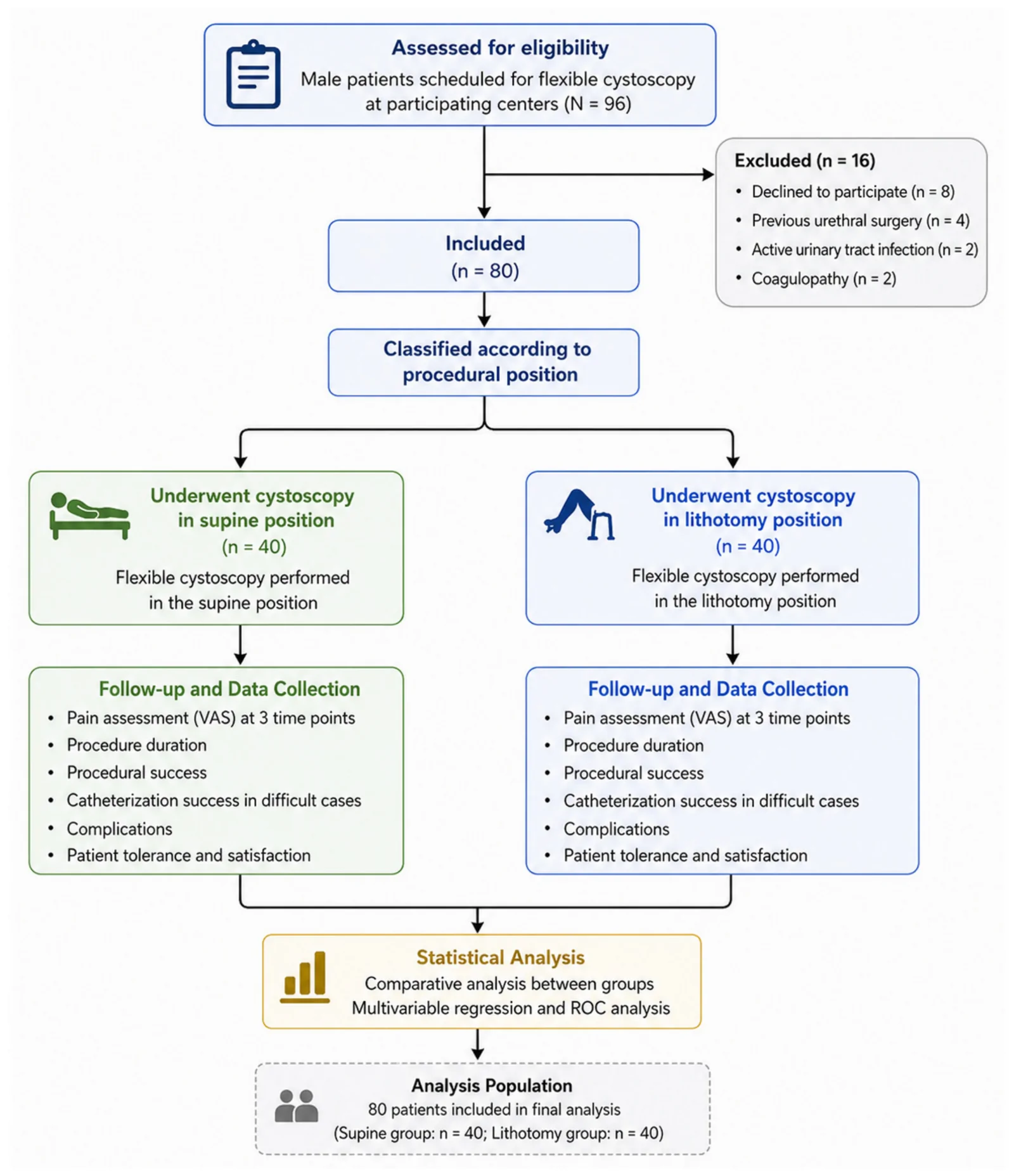
Flowchart of the Study.

The medical records of 96 adult male patients who underwent flexible cystoscopy during the study period were retrospectively screened for eligibility. Of these, 16 patients were excluded because consent for research use was not available or was declined (*n* = 8), because of previous urethral surgery (*n* = 4), active urinary tract infection at the time of the procedure (*n* = 2), or coagulopathy (*n* = 2). A total of 80 retrospectively identified patients met the eligibility criteria and were included in the analysis. Patients were classified according to the position actually used during cystoscopy, with 40 patients in the supine group and 40 patients in the lithotomy group. Flexible cystoscopy was performed in the respective positions in each group (Figure 1).

Selection-related information was variably available in the retrospective medical records. Among patients with sufficient documentation, mobility or musculoskeletal limitations were recorded more frequently in the supine group than in the lithotomy group (14/32 [43.8%] vs. 4/30 [13.3%], *p* = 0.012). Similarly, difficulty assuming or maintaining the lithotomy position was more frequent in the supine group (10/28 [35.7%] vs. 2/27 [7.4%], *p* = 0.020). The frequencies of documented preprocedural anxiety (5/26 [19.2%] vs. 4/25 [16.0%], *p* = 1.000) and previous cystoscopy experience (13/35 [37.1%] vs. 12/33 [36.4%], *p* = 1.000) were comparable between the groups. An explicit reason for position selection was documented for 29/40 patients (72.5%) in the supine group and 22/40 patients (55.0%) in the lithotomy group (*p* = 0.162). Because these variables were incompletely and non-standardly documented, the findings are presented descriptively using available-case denominators (Table 1).

A total of 80 patients were included in the study, with 40 patients in the supine group and 40 in the lithotomy group. The mean age of the study population was 61.8 ± 10.7 years, with no significant difference between the supine and lithotomy groups (60.9 ± 10.3 vs. 62.7 ± 11.1, *p* = 0.432). The mean body mass index (BMI) was 27.6 ± 3.8 kg/m^2^ in the overall cohort, with comparable values between the groups (27.3 ± 3.6 vs. 27.9 ± 4.0, *p* = 0.518). Hematuria was present in 42.5% of patients, lower urinary tract symptoms in 35.0%, and suspected urethral pathology in 22.5%, with similar distributions across both groups (all *p* > 0.05). The operator distribution was not identical between the groups. Operator A performed 26/40 procedures (65.0%) in the supine group and 18/40 procedures (45.0%) in the lithotomy group, whereas Operator B performed 14/40 (35.0%) and 22/40 (55.0%) procedures, respectively (*p* = 0.117). The prevalence of diabetes mellitus, hypertension, and coronary artery disease was 26.3%, 45.0%, and 17.5%, respectively, with no statistically significant differences between the groups (all *p* > 0.05). Anticoagulant use was observed in 23.8% of patients, and 30.0% had a history of previous urological intervention, with comparable rates between the supine and lithotomy groups (*p* = 0.795 and *p* = 0.627, respectively) (Table 1).

VAS scores were lower in the supine group during cystoscope insertion (2.1 ± 1.0 vs. 3.4 ± 1.2; mean difference: −1.30, 95% CI: −1.79 to −0.81; *p* < 0.001), bladder evaluation (1.8 ± 0.9 vs. 2.6 ± 1.1; mean difference: −0.80, 95% CI: −1.25 to −0.35; *p* = 0.001), and immediately after the procedure (1.2 ± 0.7 vs. 2.0 ± 0.9; mean difference: −0.80, 95% CI: −1.16 to −0.44; *p* < 0.001). The corresponding standardized effect sizes were Cohen’s d = 1.17 (95% CI: 0.70 to 1.64), 0.79 (95% CI: 0.33 to 1.25), and 1.00 (95% CI: 0.53 to 1.47), respectively. Mean procedure duration was 6.8 ± 2.1 min in the supine group and 7.4 ± 2.3 min in the lithotomy group (mean difference: −0.60 min, 95% CI: −1.58 to 0.38; *p* = 0.186). Procedural success occurred in 39/40 patients (97.5%) in the supine group and 38/40 patients (95.0%) in the lithotomy group (risk difference: 2.5 percentage points, 95% CI: −8.5 to 14.2; two-sided Fisher’s exact *p* = 1.000).

**Figure 2:**
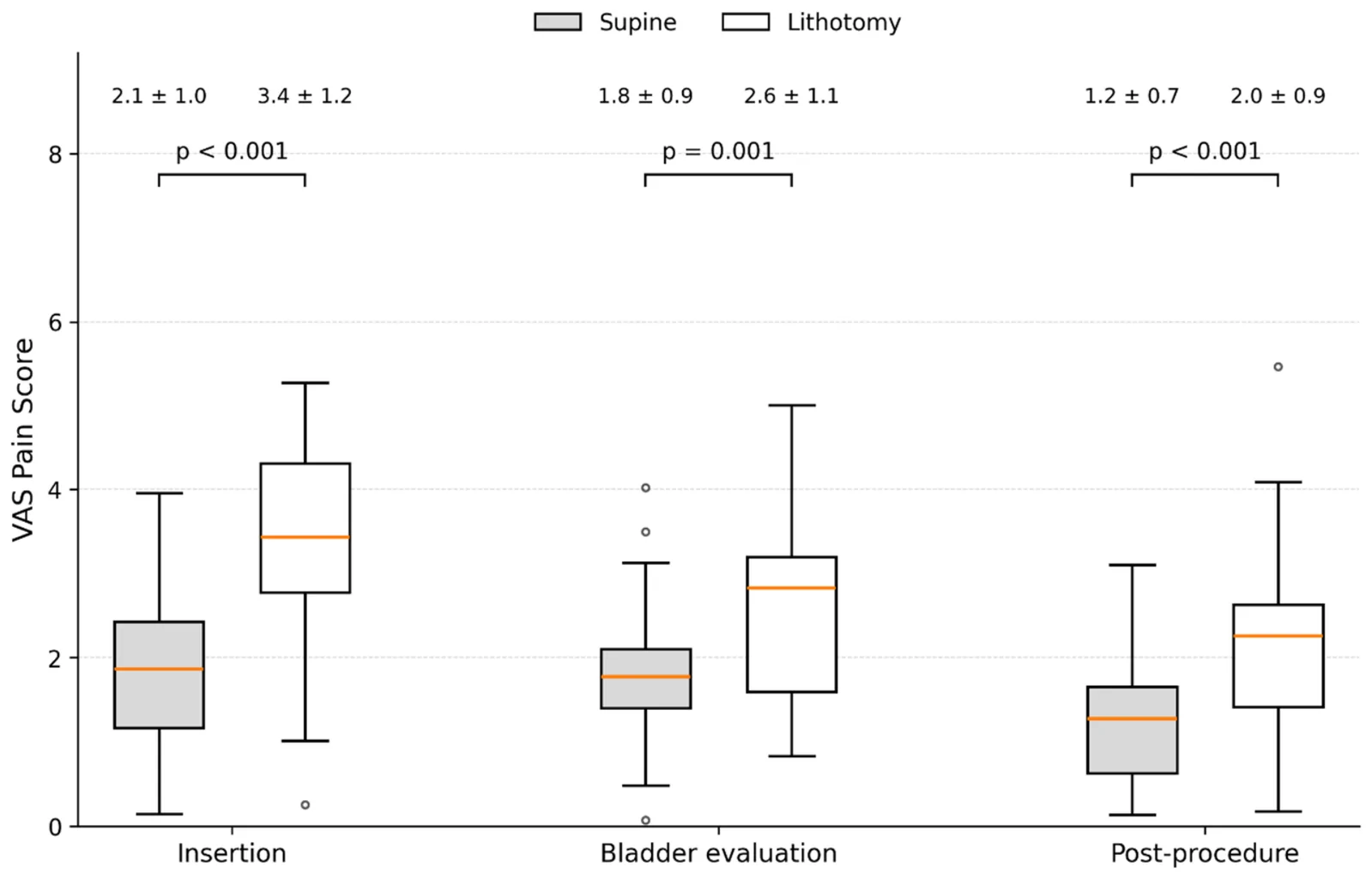
Boxplot comparison of Visual Analog Scale (VAS) pain scores between the supine and lithotomy groups during cystoscope insertion, bladder evaluation, and immediately after the procedure. The boxes represent the interquartile range (IQR), and the horizontal line within each box indicates the median. Whiskers extend to the most extreme observations within 1.5 × IQR below the first quartile and above the third quartile; observations beyond these limits are displayed individually as outliers. Values above the boxes are presented as mean ± standard deviation. Between-group *p*-values are shown for each assessment time point.

An exploratory post hoc subgroup analysis was conducted among 40 patients with documented failure of conventional blind catheterization, comprising 20 patients in the supine group and 20 in the lithotomy group. Cystoscopy-guided catheter placement was successful in 19/20 patients (95.0%) in the supine group and 13/20 patients (65.0%) in the lithotomy group (risk difference: 30.0 percentage points, 95% CI: 4.9 to 52.1; two-sided Fisher’s exact *p* = 0.044). This subgroup was not prespecified in the study protocol or included in the original sample-size calculation. Any complication occurred in 4/40 patients (10.0%) in the supine group and 7/40 patients (17.5%) in the lithotomy group (risk difference: −7.5 percentage points, 95% CI: −23.2 to 8.2; two-sided Fisher’s exact *p* = 0.518). Urethral trauma was recorded in 1/40 (2.5%) and 3/40 patients (7.5%), respectively (*p* = 0.615); hematuria requiring intervention in 1/40 (2.5%) and 2/40 (5.0%), respectively (*p* = 1.000); urinary tract infection in 2/40 (5.0%) and 3/40 (7.5%), respectively (*p* = 1.000); and acute urinary retention in 0/40 (0.0%) and 1/40 patient (2.5%), respectively (*p* = 1.000). Mean patient-reported procedural tolerance scores were 8.6 ± 1.1 and 7.2 ± 1.4 (mean difference: 1.40, 95% CI: 0.84 to 1.96; *p* < 0.001), while mean overall satisfaction scores were 8.8 ± 1.0 and 7.4 ± 1.3 (mean difference: 1.40, 95% CI: 0.88 to 1.92; *p* < 0.001), respectively. Procedural tolerance and overall satisfaction were assessed using separate single-item 0–10 rating scales (Table 2, Figure 2).

Complete data for all variables included in the primary multivariable model were available for all 80 patients; therefore, no imputation was performed. In the exploratory multivariable linear regression analysis, supine positioning was associated with a 1.21-point lower VAS score during cystoscope insertion after adjustment for age, BMI, anticoagulant use, and previous urological intervention (B = −1.21, SE = 0.27, 95% CI: −1.75 to −0.67; standardized β = −0.48; *p* < 0.001). Age, BMI, anticoagulant use, and previous urological intervention were not statistically associated with insertion-related VAS scores. The overall model was statistically significant, F(5, 74) = 6.65, *p* < 0.001, and explained 31% of the variance in insertion-related VAS scores (R^2^ = 0.31; adjusted R^2^ = 0.26) (Table 3).

Multicollinearity diagnostics did not indicate an important collinearity problem in the satisfaction model (VIF range: 1.04–1.39; tolerance range: 0.72–0.96). Complete data for all variables included in the secondary multivariable model were available for all 80 patients; therefore, no imputation was performed. In the exploratory multivariable linear regression analysis, supine positioning was associated with a 0.72-point higher overall satisfaction score after adjustment for insertion-related pain and procedure duration (B = 0.72, SE = 0.24, 95% CI: 0.24 to 1.20; standardized β = 0.28; *p* = 0.004). Each 1-point increase in the VAS score during cystoscope insertion was associated with a 0.33-point decrease in overall satisfaction (B = −0.33, SE = 0.09, 95% CI: −0.51 to −0.15; standardized β = −0.31; *p* < 0.001). Procedure duration was not statistically associated with satisfaction (B = −0.04, SE = 0.05, 95% CI: −0.14 to 0.06; standardized β = −0.07; *p* = 0.425). The overall model was statistically significant, F(3, 76) = 19.11, *p* < 0.001, and explained 43% of the variance in overall satisfaction scores (R^2^ = 0.43; adjusted R^2^ = 0.41) (Table 4).

## 4. Discussion

In this retrospective observational study, patients who underwent flexible cystoscopy in the supine position reported lower pain scores and higher tolerance and satisfaction than those who underwent the procedure in the lithotomy position. Procedural success and complication rates did not differ significantly between the groups. These findings represent unadjusted and covariate-adjusted associations within a non-randomized cohort. Because procedural position was selected by the treating urologist and was influenced by clinical feasibility, including patient mobility, the observed differences cannot be attributed solely to patient position. Moreover, comparability of the measured baseline characteristics does not exclude residual confounding by anxiety, urethral anatomy, previous cystoscopy experience, baseline positional discomfort, and other unmeasured factors. The results should therefore be interpreted as exploratory and hypothesis-generating.

Pain reduction is one of the most important determinants of patient acceptability during cystoscopy. Previous studies have consistently shown that flexible cystoscopy is associated with lower pain scores compared to rigid cystoscopy. Krajewski et al. reported that flexible cystoscopy significantly improves patient comfort, particularly in male patients undergoing repeated procedures [18]. Raskolnikov et al. demonstrated in a meta-analysis that procedural factors, including instrument type and technique, significantly influence pain perception during cystoscopy [19]. However, these studies primarily focused on instrument-related factors rather than patient positioning. Our findings add preliminary observational data showing that lower pain scores were reported among patients who underwent cystoscopy in the supine position. However, because procedural position was not randomized and relevant patient-level confounders were not systematically measured, the study cannot determine whether positioning itself caused the observed difference.

Several mechanisms may potentially explain the observed association, although these cannot be established from the present retrospective data. Lithotomy positioning requires forced hip flexion, abduction, and external rotation, which may increase discomfort, particularly in elderly patients or those with musculoskeletal limitations. Bentsen et al. highlighted that patient positioning is a critical determinant of peri-procedural discomfort and may contribute to adverse physiological and mechanical effects [20]. Vrettos et al. emphasized that positioning-related factors can influence procedural tolerance and patient experience in endourological interventions [21]. In contrast, the supine position is more physiologically neutral and avoids additional mechanical strain, which may explain the observed reduction in pain scores in our cohort.

VAS is an inherently subjective measure, and cystoscopy-related pain may be influenced by multiple patient- and procedure-related factors. Anxiety, expectations, previous cystoscopy experience, baseline discomfort, mobility limitations, urethral anatomy, prostatic obstruction, and individual pain sensitivity may affect the reported score. Operator experience, insertion speed and technique, communication during the procedure, lubrication, and the duration of local anesthetic exposure may also contribute to pain variability. Although the same cystoscope system, local anesthetic protocol, and general procedural technique were used in both groups, these measures do not eliminate patient-level or operator-related variability. Several of these factors were not systematically documented and could not be included in the adjusted analysis; therefore, residual confounding and measurement variability remain possible.

Lower insertion-related VAS scores were associated with higher overall satisfaction in the present cohort. Previous studies have similarly reported relationships between procedural discomfort and patient-reported experience during cystoscopy [13,14]. Patients in the supine group reported higher mean satisfaction scores than those in the lithotomy group. However, satisfaction was a secondary exploratory outcome, and the observed difference was not adjusted for potentially relevant patient-level confounders. However, because position selection was clinician-directed and relevant patient-level confounders were not systematically measured, these findings do not establish procedural position as an independent causal determinant of satisfaction.

A recent randomized trial comparing supine and lithotomy flexible cystoscopy in 142 male patients similarly reported lower pain scores and shorter procedure duration in the supine group [22]. The direction of the pain findings in the present study is consistent with that randomized evidence. However, unlike the randomized trial, procedural position in our cohort was selected by the treating urologist according to clinical feasibility and patient characteristics. Consequently, the present findings should not be interpreted as independent causal evidence of positional superiority. Rather, they provide complementary exploratory data on pain recorded at multiple procedural stages and on tolerance, satisfaction, complications, and procedural outcomes in routine clinical practice.

From a practical perspective, flexible cystoscopy in the supine position was performed on an examination table or hospital bed without leg elevation, lithotomy stirrups, or repositioning into lithotomy. This feature may be relevant for selected patients with restricted mobility, contractures, fractures, critical illness, or difficulty assuming the lithotomy position. However, these observations should not be interpreted as evidence that supine positioning improves patient outcomes or operational efficiency. Preparation time, staff workload, transfer requirements, workflow efficiency, and patient preference for future procedures were not directly evaluated. Therefore, any potential operational advantages of supine positioning require direct assessment in prospectively designed studies.

No statistically significant between-group differences were observed in procedural success or recorded complications. However, the small number of unsuccessful procedures and complications limits statistical precision and precludes conclusions regarding equivalence or comparative safety. Procedural success rates were high and comparable between groups, and complication rates were low without significant differences. These findings are consistent with previous reports indicating that flexible cystoscopy is a safe procedure with a low complication profile [23,24]. Beyond these findings, flexible cystoscopy offers important practical advantages due to its compact and portable design. In our clinical setting, its use allowed procedures to be performed under local anesthesia without the need for patient repositioning or operating room preparation. This approach may be particularly beneficial in patients for whom lithotomy positioning is difficult or contraindicated, such as those with musculoskeletal limitations, contractures, fractures, or critical illness. In such populations, the ability to perform cystoscopy in the supine position may facilitate both diagnostic evaluation and minor therapeutic interventions, including catheterization under direct visualization. Our findings support these practical advantages, as the supine approach was associated with high procedural success rates and improved patient tolerance.

In the exploratory post hoc subgroup of patients with documented difficult catheterization, cystoscopy-guided catheter placement was successful in 19/20 patients in the supine group and 13/20 patients in the lithotomy group. However, this analysis included only 20 patients per group, was not prespecified or separately powered, and was based on a small number of catheterization failures. It was also subject to clinician-directed position selection and confounding by indication. Consequently, the observed difference should not be interpreted as evidence that supine positioning improves catheterization success. This finding is hypothesis-generating and requires evaluation in a larger, prospectively defined and adequately powered cohort. The additional descriptive review showed that [mobility-related limitations/difficulty assuming lithotomy] were more frequently documented in the supine group, confirming that the two groups differed in at least some factors involved in position selection. The direction of the resulting bias cannot be determined with certainty. Greater baseline physical limitation could have increased discomfort in the supine group and biased the association toward the null; conversely, clinician selection of the position considered more tolerable for an individual patient could have favored lower pain scores in that group. Moreover, the incomplete and non-standardized documentation of anxiety and previous cystoscopy experience prevented reliable adjustment for these factors.

The findings of this study have several clinical implications. The observed associations suggest that supine flexible cystoscopy may warrant consideration when lithotomy positioning is difficult or clinically impractical. However, the present findings are insufficient to establish the superiority of one position or to support routine changes in clinical practice. Performing flexible cystoscopy without leg elevation or lithotomy stirrups may have practical value in selected patients; however, preparation time, staff workload, and workflow efficiency were not directly measured in this study. The observed associations may inform the design of future prospective studies; however, the present data are insufficient to support a prediction tool or position-based clinical decision rule.

This study has several important limitations. First, its retrospective, non-randomized observational design precludes causal inference. Procedural position was selected by the treating urologist according to clinical feasibility, and patients with limited mobility or difficulty assuming lithotomy were more likely to undergo cystoscopy in the supine position. This clinician-directed selection introduces selection bias and confounding by indication. The distributions of study center and primary operator also showed modest between-group differences. Although these differences were not statistically significant, operator preference, procedural technique, and center-specific practice patterns may have influenced both position selection and patient-reported outcomes. The limited sample size prevented robust operator-stratified or center-stratified adjustment; therefore, residual operator- and center-level confounding cannot be excluded. Because operator preferences and institutional positioning practices may vary across clinical settings, the present findings may not be generalizable to institutions with different patient-selection approaches, staffing structures, operator experience, or procedural-positioning protocols. Although the groups were comparable with respect to the measured demographic and clinical characteristics, mobility limitation, baseline position-related discomfort, patient anxiety, detailed urethral anatomy, and previous cystoscopy experience were not systematically quantified and could not be included in the adjusted analysis. Therefore, substantial residual confounding cannot be excluded, and the observed differences in pain, tolerance, and satisfaction cannot be attributed solely to procedural position. The relatively small sample size limits the precision and stability of the estimates, and fitting several predictors in a cohort of 80 patients may have increased the risk of model overfitting. Accordingly, the adjusted estimates and overall findings should be interpreted cautiously and considered exploratory and hypothesis-generating rather than practice-changing; confirmation in adequately powered prospective randomized studies is required.

Second, the study included only adult male patients; therefore, the findings cannot be generalized to women undergoing flexible cystoscopy because of differences in urethral anatomy, instrument insertion, and procedural experience. Third, the difficult catheterization analysis was an exploratory post hoc analysis involving only 40 patients, with 20 patients in each positional group. It was not prespecified or separately powered and was based on a small number of catheterization failures. Accordingly, this subgroup analysis was severely underpowered for reliable comparative inference and should be interpreted solely as hypothesis-generating; it remains vulnerable to random error, misclassification, selection bias, and confounding by indication. Fourth, procedural tolerance and overall satisfaction were assessed using separate single-item rating scales rather than validated multidimensional instruments. Although tolerance was intended to represent the patient’s ability to endure the procedure and satisfaction reflected a broader evaluation of the procedural experience, some conceptual overlap remains possible. Furthermore, because insertion-related pain may lie on the pathway between procedural position and overall satisfaction, the secondary satisfaction model estimates conditional rather than total associations and should not be interpreted as evidence of a causal effect. In addition, procedural position was directly observable, and neither patients nor the clinical personnel recording the patient-reported outcomes were blinded. Because the scores were obtained during routine clinical care rather than under a research-specific blinded assessment protocol, response bias, observer-related measurement variability, and social-desirability bias cannot be excluded. Finally, all procedures were performed using a single flexible cystoscope system. Although this standardized instrumentation across the groups, device-specific differences in shaft diameter and stiffness, distal-tip flexibility, surface characteristics, working-channel dimensions, and optical performance may limit generalizability to other single-use or reusable flexible cystoscope platforms.

## 5. Conclusions

In this retrospective, non-randomized observational study of adult male patients, clinician-selected supine positioning during flexible cystoscopy was associated with lower reported pain and higher procedural tolerance and overall satisfaction compared with lithotomy positioning. No statistically significant between-group differences were observed in overall procedural success or complication rates. Supine positioning was technically feasible on an examination table or hospital bed without leg elevation, lithotomy stirrups, or repositioning; however, the present study did not establish a patient-level or operational benefit of this approach. Clinician-directed position selection, differences in mobility-related characteristics, and residual unmeasured confounding preclude causal inference. Accordingly, these findings should be considered exploratory and hypothesis-generating. They should primarily inform the design of adequately powered prospective randomized studies rather than immediate changes in routine clinical practice.

## Figures and Tables

**Table 1:** Baseline demographic, clinical, procedural-selection, center, and operator characteristics of the study population.

Variable	Total (*n* = 80)	Supine Group (*n* = 40)	Lithotomy Group (*n* = 40)	*p*-Value
Age (years)	61.8 ± 10.7	60.9 ± 10.3	62.7 ± 11.1	0.432
BMI (kg/m^2^)	27.6 ± 3.8	27.3 ± 3.6	27.9 ± 4.0	0.518
Hematuria, *n* (%)	34 (42.5%)	18 (45.0%)	16 (40.0%)	0.651
Study center, *n* (%)				0.263
NCR International Hospital	42 (52.5%)	24 (60.0%)	18 (45.0%)	
Abdulkadir Yuksel Hospital	38 (47.5%)	16 (40.0%)	22 (55.0%)	
Primary operator, *n* (%)				0.117
Operator A	44 (55.0%)	26 (65.0%)	18 (45.0%)	
Operator B	36 (45.0%)	14 (35.0%)	22 (55.0%)	
Lower urinary tract symptoms, *n* (%)	28 (35.0%)	13 (32.5%)	15 (37.5%)	0.634
Suspected urethral pathology, *n* (%)	18 (22.5%)	9 (22.5%)	9 (22.5%)	1.000
Diabetes mellitus, *n* (%)	21 (26.3%)	10 (25.0%)	11 (27.5%)	0.799
Hypertension, *n* (%)	36 (45.0%)	17 (42.5%)	19 (47.5%)	0.655
Coronary artery disease, *n* (%)	14 (17.5%)	6 (15.0%)	8 (20.0%)	0.556
Anticoagulant use, *n* (%)	19 (23.8%)	10 (25.0%)	9 (22.5%)	0.795
Previous urological intervention, *n* (%)	24 (30.0%)	11 (27.5%)	13 (32.5%)	0.627
Position-selection characteristics				
Documented mobility or musculoskeletal limitation, *n*/*N* available (%)	18/62 (29.0%)	14/32 (43.8%)	4/30 (13.3%)	0.012
Documented difficulty assuming or maintaining lithotomy, *n*/*N* available (%)	12/55 (21.8%)	10/28 (35.7%)	2/27 (7.4%)	0.020
Documented preprocedural anxiety, *n*/*N* available (%)	9/51 (17.6%)	5/26 (19.2%)	4/25 (16.0%)	1.000
Previous cystoscopy experience, *n*/*N* available (%)	25/68 (36.8%)	13/35 (37.1%)	12/33 (36.4%)	1.000
Explicit reason for position selection documented, *n* (%)	51 (63.8%)	29 (72.5%)	22 (55.0%)	0.162

Selection-related variables were abstracted during a secondary review of the source records. Because these factors were not collected using standardized forms, values are presented as *n*/*N* available (%), where *N* represents the number of patients with sufficient documentation for the relevant variable. Lack of documentation was classified as missing rather than as absence of the characteristic. Between-group comparisons were exploratory and were performed using Fisher’s exact test. Continuous variables are presented as mean ± standard deviation, and categorical variables as *n* (%), unless otherwise indicated. Overall distributions of study center and operator were compared using Pearson’s chi-square test or Fisher’s exact test, as appropriate. Operators were anonymized as A–B. Selection-related variables with incomplete documentation are presented as *n*/*N* available (%), with undocumented observations treated as missing rather than as absence of the characteristic. BMI, body mass index.

**Table 2:** Comparison of procedural and clinical outcomes.

Variable	Supine Group (*n* = 40)	Lithotomy Group (*n* = 40)	*p*-Value	Effect Estimate (95% CI)
VAS score—insertion	2.1 ± 1.0	3.4 ± 1.2	<0.001	MD −1.30 (−1.79 to −0.81)
VAS score—bladder evaluation	1.8 ± 0.9	2.6 ± 1.1	0.001	MD −0.80 (−1.25 to −0.35)
VAS score—post-procedure	1.2 ± 0.7	2.0 ± 0.9	<0.001	MD −0.80 (−1.16 to −0.44)
Procedure duration (min)	6.8 ± 2.1	7.4 ± 2.3	0.186	MD −0.60 min (−1.58 to 0.38)
Procedural success, *n* (%)	39 (97.5%)	38 (95.0%)	1.000	RD 2.5 percentage points (−8.5 to 14.2)
Exploratory post hoc subgroup: successful cystoscopy-guided catheterization among patients with documented difficult catheterization, *n*/*N* (%) *	19/20 (95.0%)	13/20 (65.0%)	0.044	RD 30.0 percentage points (4.9 to 52.1)
Any complication, *n* (%)	4 (10.0%)	7 (17.5%)	0.518	RD −7.5 percentage points (−23.2 to 8.2)
Urethral trauma, *n* (%)	1 (2.5%)	3 (7.5%)	0.615	RD −5.0 percentage points (−17.5 to 6.5)
Hematuria requiring intervention, *n* (%)	1 (2.5%)	2 (5.0%)	1.000	RD −2.5 percentage points (−14.2 to 8.5)
Urinary tract infection, *n* (%)	2 (5.0%)	3 (7.5%)	1.000	RD −2.5 percentage points (−15.4 to 10.0)
Acute urinary retention, *n* (%)	0 (0.0%)	1 (2.5%)	1.000	RD −2.5 percentage points (−12.9 to 6.5)
Patient-reported procedural tolerance score (0–10)	8.6 ± 1.1	7.2 ± 1.4	<0.001	MD 1.40 (0.84 to 1.96)
Overall patient satisfaction score (0–10)	8.8 ± 1.0	7.4 ± 1.3	<0.001	MD 1.40 (0.88 to 1.92)

* The difficult catheterization analysis was exploratory and post hoc and included 40 patients, with 20 patients in each positional group. It was not prespecified or separately powered. The *p*-value was calculated using a two-sided Fisher’s exact test; the finding should be considered hypothesis-generating. This subgroup was not included in the original sample-size calculation. Procedural tolerance and overall satisfaction were assessed using separate single-item 0–10 rating scales; higher scores indicate greater tolerance or satisfaction, respectively. These exploratory scales were not specifically validated for flexible cystoscopy. CI: Confidence Intervals; MD: Mean Difference; RD: Risk Difference.

**Table 3:** Complete exploratory multivariable linear regression model for VAS score during cystoscope insertion.

Variable	Unstandardized β	SE	95% CI	Standardized β	*p*-Value
Constant	1.72	0.78	0.17 to 3.27	—	0.031
Supine position	−1.21	0.27	−1.75 to −0.67	−0.48	<0.001
Age, per 1-year increase	0.01	0.01	−0.01 to 0.03	0.09	0.284
BMI, per 1 kg/m^2^ increase	0.03	0.02	−0.02 to 0.08	0.11	0.221
Anticoagulant use	0.42	0.24	−0.05 to 0.89	0.16	0.078
Previous urological intervention	0.31	0.22	−0.12 to 0.74	0.12	0.156
Model summary: *n* = 80; R^2^ = 0.31; adjusted R^2^ = 0.26; F(5, 74) = 6.65; *p* < 0.001.					

The dependent variable was the VAS score during cystoscope insertion, analyzed as a continuous 0–10 outcome. All covariates were entered simultaneously. Procedural position was coded as supine = 1 and lithotomy = 0; anticoagulant use and previous urological intervention were coded as yes = 1 and no = 0. Accordingly, a negative B for the supine position indicates a lower adjusted mean VAS score relative to lithotomy positioning. Complete covariate data were available for all 80 patients; therefore, no imputation was performed. The model was exploratory and was restricted to clinically relevant covariates available in the retrospective dataset. B, unstandardized regression coefficient; β, standardized regression coefficient; BMI, body mass index; CI, confidence interval; SE, standard error; VAS, Visual Analog Scale.

**Table 4:** Complete exploratory multivariable linear regression model for overall patient satisfaction score.

Variable	Unstandardized β	SE	95% CI	Standardized β	*p*-Value
Constant	8.93	0.52	7.89 to 9.97	—	<0.001
Supine position	0.72	0.24	0.24 to 1.20	0.28	0.004
VAS score during cystoscope insertion, per 1-point increase	−0.33	0.09	−0.51 to −0.15	−0.31	<0.001
Procedure duration, per 1 min increase	−0.04	0.05	−0.14 to 0.06	−0.07	0.425
Model fit: *n* = 80; R^2^ = 0.43; adjusted R^2^ = 0.41; F(3, 76) = 19.11; *p* < 0.001.					

The dependent variable was overall patient satisfaction measured on a continuous 0–10 scale, with higher scores indicating greater satisfaction. All predictors were entered simultaneously. Procedural position was coded as supine = 1 and lithotomy = 0. Accordingly, a positive B for the supine position indicates a higher adjusted mean satisfaction score relative to lithotomy positioning. VAS was measured on a 0–10 scale, with higher scores indicating greater pain. Complete data for all variables included in the model were available for all 80 patients; therefore, no imputation was performed. The model was exploratory and was not intended to establish causal or mediating relationships. B, unstandardized regression coefficient; β, standardized regression coefficient; CI, confidence interval; SE, standard error; VAS, Visual Analog Scale.

## Data Availability

The datasets generated and/or analyzed during the current study are not publicly available due to patient privacy, confidentiality, and ethical restrictions, as the data contain potentially identifiable clinical information. De-identified data may be made available from the corresponding author upon reasonable request and with appropriate ethical approval.

## Abbreviations

BMI	Body Mass Index
CI	Confidence Interval
IQR	Interquartile Range
OR	Odds Ratio
SD	Standard Deviation
VAS	Visual Analog Scale

## References

[1] Devlies W., de Jong J.J., Hofmann F., Bruins H.M., Zuiverloon T.C.M., Smith E.J., Yuan Y., van Rhijn B.W.G., Mostafid H., Santesso N. (2024). The Diagnostic Accuracy of Cystoscopy for Detecting Bladder Cancer in Adults Presenting with Haematuria: A Systematic Review from the European Association of Urology Guidelines Office. Eur. Urol. Focus.

[2] Taneja R., Pandey S., Priyadarshi S., Goel A., Jain A., Sharma R., Purohit N., Bandukwalla V., Tanvir, Ragavan M. (2023). Diagnostic and therapeutic cystoscopy in bladder pain syndrome/interstitial cystitis: Systematic review of literature and consensus on methodology. Int. Urogynecol. J..

[3] Waisbrod S., Natsos A., Wettstein M.S., Saba K., Hermanns T., Fankhauser C.D., Müller A. (2021). Assessment of Diagnostic Yield of Cystoscopy and Computed Tomographic Urography for Urinary Tract Cancers in Patients Evaluated for Microhematuria. JAMA Netw. Open.

[4] Ben-David R., Morgan S., Savin Z., Dekalo S., Sofer M., Beri A., Yossepowitch O., Mano R. (2022). Flexible Cystoscopy in the Setting of Macroscopic Hematuria: Do the Findings Justify Its Use?. Urol. Int..

[5] Horsburgh B.A., Higgins M. (2016). A Study of Occupational Radiation Dosimetry During Fluoroscopically Guided Simulated Urological Surgery in the Lithotomy Position. J. Endourol..

[6] Kasap Y., Senel S., Uzun E., Polat M.E., Koudonas A., Ozden C. (2022). Does surgical position affect infective complications in percutaneous nephrolithotomy?. Urolithiasis.

[7] Ginsburg K.B., Pape K., Heilbronn C., Levin M., Cher M.L. (2017). Prospective assessment of positioning-related pain in robotic urologic surgery. J. Robot. Surg..

[8] Zillioux J., Krupski T. (2017). Patient positioning during minimally invasive surgery: What is current best practice?. Robot. Surg. Res. Rev..

[9] Larsen C.H., Christensen S.M., Parwaei E., Thomsen T., Thomsen F.F. (2025). Incidence and location of positioning-related injuries in lateral positioning during laparoscopic kidney surgery. Int. Urol. Nephrol..

[10] Greenberg J.W., Lai W.R., Sanekommu G., Thomas R. (2025). Complications and Management of Robotic Lower Urinary Tract Procedures. The Comprehensive Atlas of Robotic Urologic Surgery.

[11] Kölükçü E., Özbek L.M., Ceylan S.R. (2019). The efficacy of Intraurethral Lidocaine Gel Use on Pain Control in Male Cystoscopy Patients. Int. J. Tokat Med. Sci..

[12] Casteleijn N.F., Vriesema J.L., Stomps S.P., van Balen O.L.W.B., Cornel E.B. (2017). The effect of office based flexible and rigid cystoscopy on pain experience in female patients. Investig. Clin. Urol..

[13] Ucer O., Temeltas G., Gumus B., Muezzinoglu T. (2020). Comparison of pain, quality of life, lower urinary tract symptoms and sexual function between flexible and rigid cystoscopy in follow-up male patients with non muscle invasive bladder cancer: A randomized controlled cross section single blind study. Int. J. Clin. Pract..

[14] Beyatli M., Gungor H.S., Inkaya A., Sobay R., Evci M.U., Tahra A., Kucuk E.V. (2026). Flexible Cystoscopy Patient Experience: Comparative Effects of Irrigation Techniques on Comfort and Satisfaction. Med. Bull. Haseki.

[15] Babjuk M., Burger M., Capoun O., Cohen D., Compérat E.M., Dominguez Escrig J.L., Gontero P., Liedberg F., Masson-Lecomte A., Mostafid A.H. (2022). European Association of Urology Guidelines on Non–muscle-invasive Bladder Cancer (Ta, T1, and Carcinoma in Situ). Eur. Urol..

[16] Hamamoto S., Okada S., Inoue T., Taguchi K., Kawase K., Okada T., Chaya R., Hattori T., Okada A., Matsuda T. (2021). Comparison of the safety and efficacy between the prone split-leg and Galdakao-modified supine Valdivia positions during endoscopic combined intrarenal surgery: A multi-institutional analysis. Int. J. Urol..

[17] Desai A.P., Smith A.B., Lotan Y. (2025). Guidance for avoiding patient pain and discomfort during transurethral cystoscopy. Expert Rev. Med. Devices.

[18] Krajewski W., Zdrojowy R., Wojciechowska J., Kościelska K., Dembowski J., Matuszewski M., Tupikowski K., Małkiewicz B., Kołodziej A. (2016). Patient comfort during flexible and rigid cystourethroscopy. Videosurgery Other Miniinvasive Tech..

[19] Raskolnikov D., Brown B., Holt S.K., Ball A.L., Lotan Y., Strope S., Schroeck F., Ullman R., Lipman R., Smith A.B. (2019). Reduction of Pain during Flexible Cystoscopy: A Systematic Review and Meta-Analysis. J. Urol..

[20] Bentsen S.B., Eide G.E., Wiig S., Rustøen T., Heen C., Bjøro B. (2024). Patient positioning on the operating table and patient safety: A systematic review and meta-analysis. J. Adv. Nurs..

[21] Vrettos T., Martinez B.B., Tsaturyan A., Liourdi D., Al-Aown A., Lattarulo M., Liatsikos E., Kallidonis P. (2023). Effect of patient positioning on anesthesiologic risk in endourological procedures. Urol. Ann..

[22] Rojanapaitoon V., Saksirisampant P. (2025). Comparison of Pain during Flexible Cystoscopy between Supine and Lithotomy Position in Male Patients: A Randomized Controlled Trial. Urol. Int..

[23] Safiullah S., Lama D.J., Patel R., Clayman R.V. (2018). Procedural Module: Flexible Cystoscopy. J. Endourol..

[24] Zorzi F., Traunero F., Jahrreiss V., Rossin G., Piasentin A., Cai T., Umari P., Liguori G., Somani B., Pietropaolo A. (2025). Office-Based Ureteral Stenting Using a Single-Use Flexible Cystoscope Under Local Anesthesia: A Two-Center Prospective Study on Feasibility and Patient Experience. J. Endourol..

